# A High-Accuracy RC Time Constant Auto-Tuning Scheme for Integrated Continuous-Time Filters

**DOI:** 10.3390/mi15010166

**Published:** 2024-01-22

**Authors:** Gang Jin, Hao Wu, Yue Yin, Lei Zheng, Yiqi Zhuang

**Affiliations:** 1 School of Microelectronic, Xidian University, Xi’an 710071, China; gjin@xidian.edu.cn (G.J.); yqzhuang@xidian.edu.cn (Y.Z.); 2 School of Computer Science, Xi’an Shiyou University, Xi’an 710071, China; 3 School of Microelectronics, Northwestern Polytechnical University, No. 127 West Youyi Road, Xi’an 710072, China; yinyue@nwpu.edu.cn; 4 Guangzhou Quanshengwei Information Technology Co., Ltd., No. 18 Kexue Avenue, Huangpu District, Guangzhou 510700, China; 13201569159@163.com

**Keywords:** CMOS circuit design, RC time constant, auto-tuning, active-RC filter

## Abstract

The reliability of the resistor-capacitor (RC) time constant of a continuous-time (CT) filter has long been an obstacle with integrated circuits. Due to process and temperature variations in complementary metal-oxide semiconductor (CMOS) technology, the absolute value of the RC time constant may vary over ±50%, which is a big issue for many integrated continuous-time analog circuits. This study proposes an on-chip RC time constant auto-tuning scheme. The proposed scheme is based on the discrete master–slave auto-tuning concept. Considering the limitations in conventional works, a higher tuning accuracy is achieved by adopting two techniques: firstly, parasitic capacitance cancelation is proposed to eliminate the effects caused by parasitic capacitance; secondly, symmetric comparison is introduced to minimize the influence of the DC offset of the comparator. A successive approximation procedure is applied to improve the tuning speed. The proposed auto-tuning scheme has been validated in 55 nm CMOS technology with a fourth-order active-RC low-pass filter under PVT variations and 60 mV input offset voltage. The average tuning error is 2.21%, and the maximum error is 3.67%. The tuning error of the proposed scheme is considerably lower than the conventional scheme.

## 1. Introduction

The accuracy of the RC time constant, which is critical for many continuous-time analog circuits, has been a common challenge in integrated devices. For example, active-RC filters are widely used in integrated circuits because of their high linearity, and the cut-off frequency of the active-RC filter is determined by the value of the RC time constant [1,2,3,4]. However, in complementary metal-oxide semiconductor (CMOS) technologies, the absolute value of resistance and capacitance is greatly affected by process and temperature. As a result, RC time constant uncertainty of up to 50% could occur due to variations in process, voltage, and temperature (PVT); this uncertainty will reduce the frequency performance of filters, leading to reliability issues in integrated circuits [5,6,7,8,9,10]. Accordingly, many real-time on-chip automatic tuning methods have been proposed and developed to improve the accuracy in the calibration of the RC time constant [2,6,11,12,13,14,15]. However, most existing methods are limited by some disadvantages. In [11,12,13,14,16], the linearity in the circuit to be tuned is compromised. In fact, high linearity is often very important in many cases [15,17]. The master–slave scheme is widely used to achieve auto-tuning of the RC time constant without interrupting the process of the system and preserve linearity [18,19,20,21,22,23,24,25]. In this scheme, similar capacitors and resistors are employed in the filter to be tuned and the tuning module. In the master tuning circuit, the value of either capacitance or resistance is adjusted discretely, under the “charge comparison” concept. When the RC time constant in the master circuit is adjusted to a desired value, the tuning process is completed, and the capacitors or resistors in the slave filter are altered accordingly. Since both circuits suffer from the same PVT variations, the RC time constant of the slave circuit can be tuned [15,19,26,27,28]. The change in capacitance does not influence the operating points of transistors; thus, the linearity of the system is not affected [6,15,29]. Unfortunately, the master–slave tuning scheme usually suffers from nonidealities in real applications. In this scheme, a node connected to capacitors and resistors is usually charged for a specific period. Since parasitic capacitances could be induced at the charged node, the accuracy of the tuning result could be reduced. Moreover, a comparator is often needed in tuning circuits, but the DC offset of the comparator could induce considerable error [30,31]. The literature shows that a conventional master–slave tuning scheme works well with less critical applications. However, the story could be different when critical applications are considered or scaled technologies are used.

In this paper, an auto-tuning scheme is proposed to achieve high tuning accuracy in RC time-constant calibration. The influence of parasitic capacitance is avoided by using the parasitic capacitance cancelation technique. The error induced by the DC offset in the comparator is minimized by using the symmetric comparison technique. Successive approximation register (SAR) logic is adopted to accelerate the tuning process. The conventional method is discussed in Section 2. Section 3 describes the architecture, working principle, and advantages of the proposed scheme. The structure is designed in a 55 nm CMOS process as well as a fourth-order active-RC low-pass filter, and the simulation results are reported in Section 4. Conclusions are drawn in Section 5.

## 2. Conventional Master–Slave Tuning Scheme

### 2.1. Active-RC Filter Performance under PVT Variation

**Figure 1:**
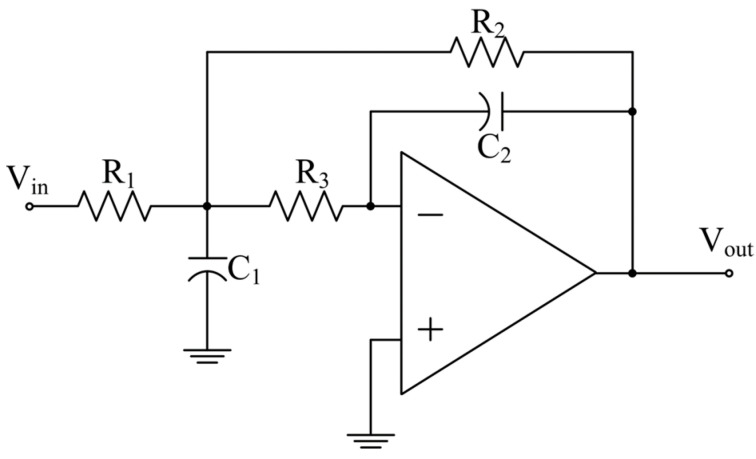
MFB low-pass biquad.

Figure 1 shows a low-pass multiple-feedback (MFB) biquad circuit, which is widely used in active-RC filters, as an example. The transfer function of this biquad is

(1) $$H\left(s\right)=\frac{\frac{1}{R_{1}R_{3}C_{1}C_{2}}}{s^{2}+\frac{1}{C_{1}}\left(\frac{1}{R_{1}}+\frac{1}{R_{2}}+\frac{1}{R_{3}}\right)s+\frac{1}{R_{2}R_{3}C_{1}C_{2}}}$$

The cut-off frequency *f*_c_ can be expressed as

(2) $$f_{c}=\frac{1}{2\pi \sqrt{R_{2}R_{3}C_{1}C_{2}}}$$

It can be seen that the cut-off frequency is determined by the RC time constant. However, the absolute values of resistors and capacitors may vary by 50% due to PVT variations, which is unacceptable in most applications. A common solution is to employ an on-chip auto-tuning circuit as an auxiliary circuit of the filter system.

### 2.2. The Classic Master–Slave Auto-Tuning Scheme

**Figure 2:**
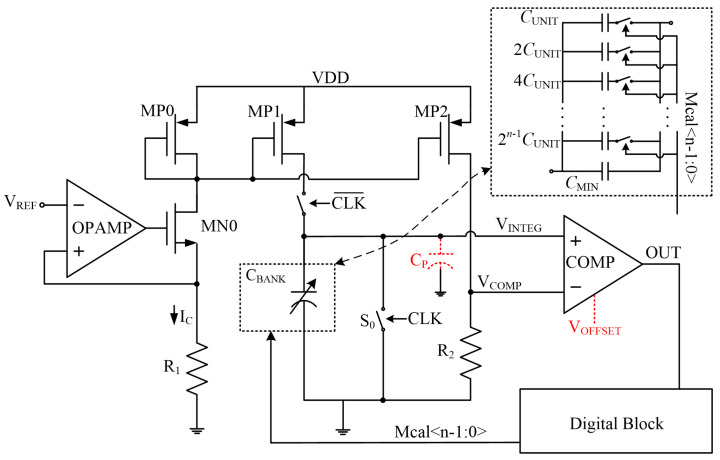
The conventional master–slave tuning scheme.

The master–slave auto-tuning scheme has been explored by previous works. In this scheme, similar capacitors and resistors are employed in the filter to be tuned and the tuning module. Figure 2 shows the master circuit of a conventional auto-tuning method [15,18]. The circuit is composed of four parts: a current bias circuit, an integrator circuit, a comparator, and a digital block. The capacitor bank and the resistor *R*_1_ are identical to those deployed in slave circuits. The capacitor bank *C_BANK_* is controlled by an *n*-bit binary control word *Mcal*<n-1:0>, as shown in Figure 2. The capacitance value of *C_BANK_* can be expressed as

(3) $$C_{BANK}=C_{MIN}+mC_{UNIT}$$

where *m* is the decimal form of *Mcal*<n-1:0>, *C_MIN_* is a fixed capacitance and *C_UNIT_* is the unit capacitance of the capacitor bank. A fixed current *I_C_* is generated by *V_REF_* and mirrored by transistors *MP*1 and *MP*2. The ratio of *R*_1_ to *R*_2_ is designed to set *V_COMP_* to a proper target voltage and is insensitive to process variations. *I_C_* and *V_COMP_* can be expressed as follows:

(4) $$I_{C}=\frac{V_{REF}}{R_{1}}$$

(5) $$V_{COMP}=V_{REF}\times \frac{R_{2}}{R_{1}}$$

The digital block generates the clock signal *CLK* whose period is *T* with a half-period at a high level. When *CLK* is high, the structure is in reset mode, where capacitor banks are discharged by *S*_0_ and *V_INTEG_* is pulled to 0. Once the negative edge of *CLK* comes, *C_BANK_* is charged by current *I_C_*. The charging process finishes after a duration of *T*/2. Ideally, the value of *V_INTEG_* after each charging is inversely proportional to *C_BANK_*:

(6) $$V_{INTEG}=\frac{I_{C}\times 0.5T}{C_{BANK}}=\frac{V_{REF}\times T}{2\times R_{1}\times C_{BANK}}$$

At the positive edge of *CLK*, a comparison between *V_INTEG_* and *V_COMP_* is performed by the comparator before the structure turns into reset mode. *Mcal*<4:0> is increased or decreased after each comparison according to the comparison result *OUT*, making the value of *V_INTEG_* approach *V_COMP_*. The following equations can be deduced when *V_INTEG_* equals *V_COMP_*:

(7) $$R_{1}C_{BANK}=\frac{T}{2}\times \frac{V_{REF}}{V_{COMP}}=\frac{k}{2}T$$

(8) $$k=\frac{V_{REF}}{V_{COMP}}=\frac{R_{1}}{R_{2}}$$

where *k* is the ratio of *R*_1_ to *R*_2_. Since the resistors are formed by the same unity polysilicon resistor patterns in the layout, the resistance variations induced by PVT variation will be identical. Consequently, the ratio *k* between the resistors is not sensitive to PVT variations. Equation (7) indicates that *R*_1_*C_BANK_* can be tuned into a fixed target value determined by *k* and *T*. The resultant control word is latched to control the capacitor bank in slave circuits.

### 2.3. Discussion on Nonidealities

The tuning accuracy of the conventional scheme suffers from nonidealities. Firstly, in the master tuning circuit, parasitic capacitance could be induced at the charged node by the comparator or the current source, inducing inherent error. Secondly, as the integrated voltage is compared with a reference voltage, the DC offset in the comparator could also induce considerable error [30,31]. Assume that parasitic capacitance at the charged node *C_P_* and offset voltage of the comparator *V_OFFSET_* are involved. Equation (7) should be rewritten as

(9) $$R_{1}\left(C_{BANK}+C_{P}\right)=\frac{T}{2}\times \frac{V_{REF}}{V_{COMP}\pm V_{OFFSET}}$$

A difference between the time constant after calibration and the ideal time constant is induced:

(10) $$\Delta RC=\left(k-\frac{V_{REF}}{V_{COMP}\pm V_{OFFSET}}\right)\times \frac{T}{2}-R_{1}C_{P}$$

In an application where the desired *C_BANK_* is 6 pF and *V_OFFSET_* is 0, according to Equation (10), a 0.5 pF parasitic capacitance could cause an error of 8.3% in the time constant; if *V_REF_* and *V_COMP_* are both 600 mV, which means *k* is 1, and *C_P_* is 0, a 50 mV input offset voltage could cause an error of 9.1% in the time constant. These errors would be unacceptable in designs with high accuracy requirements, and even greater error could be caused when these nonidealities occur at the same time.

## 3. Proposed Method

**Figure 3:**
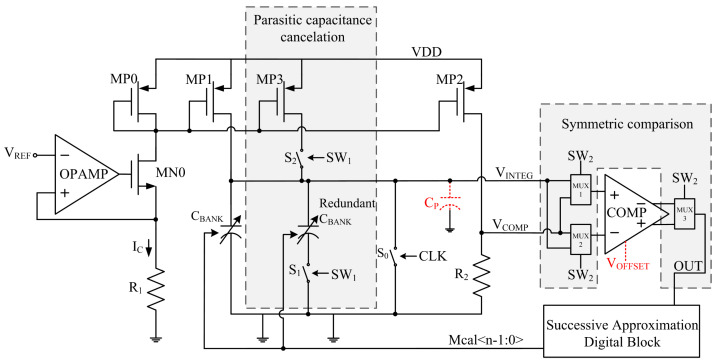
The proposed master–slave scheme.

The basic architecture of the proposed tuning scheme is shown in Figure 3. The proposed method utilizes two techniques to minimize the nonidealities discussed above, namely parasitic capacitance cancelation and symmetric comparison, and improve the auto-tuning accuracy:

1. Parasitic capacitance cancelation technique:

As can be seen in Figure 3, two identical capacitor banks are implemented. The redundant *C_BANK_* is controlled by a switch *S*_1_. A redundant current source *MP*3 is implemented as well. The digital signal *SW*_1_ controls these redundant elements through switches *S*_1_ and *S*_2_. Firstly, the RC time constant is calibrated with *SW*_1_ being 0, which means the redundant elements are not involved in charging *V_INTEG_*. The control word *m_a_* obtained in the first calibration could satisfy Equation (11):

(11) $$C_{MIN}+m_{a}C_{UNIT}+C_{P}=\frac{I_{C}\times 0.5T}{V_{COMP}}$$

where *m_a_* is the decimal form of the binary control word *Mcal*<n-1:0>. Secondly, the time constant is calibrated again with *SW*_1_ being 1; the redundant *C_BANK_* and *MP*3 are included in charge node *V_INTEG_*. The obtained control word *m_b_* could satisfy Equation (12):

(12) $$2\left(C_{MIN}+m_{b}C_{UNIT}\right)+C_{P}=\frac{2I_{C}\times 0.5T}{V_{COMP}}$$

By subtracting Equation (11) from Equation (12), the parasitic capacitance *C_P_* could be eliminated:

(13) $$C_{MIN}+\left(2m_{b}-m_{a}\right)C_{UNIT}=\frac{I_{C}\times 0.5T}{V_{COMP}}$$

In fact, the target control word *m_TAGT_* is supposed to make *V_INTEG_* equal *V_COMP_* after being charged for a duration of *T*⁄2 without *C_P_*, so *m_TAGT_* should satisfy Equation (14):

(14) $$C_{MIN}+m_{TAGT}C_{UNIT}=\frac{I_{C}\times 0.5T}{V_{COMP}}$$

As a result, the value of the target control word *m_TAGT_* can be deduced from Equations (13) and (14):

(15) $$m_{TAGT}=2m_{b}-m_{a}$$

which is able to make *V_INTEG_* equal *V_COMP_* after being charged for a duration of T2 without the presence of *C_P_*.

2. Symmetric comparison technique:

As can be seen in Figure 3, three multiplexers controlled by the digital signal *SW*_2_ are placed at the input ports and output ports of the comparator, respectively. When *SW*_2_ is 0, the multiplexers pass *V_COMP_* to the negative input of the comparator and *V_INTEG_* to the positive input. The positive output of the comparator is sampled as *OUT*. After an auto-tuning procedure, the obtained control word *m_x_* could satisfy Equation (16):

(16) $$C_{MIN}+m_{x}C_{UNIT}=\frac{I_{C}\times 0.5T}{V_{COMP}+V_{OFFSET}}$$

When *SW*_2_ is 1, the input ports of the comparator are reversed by the multiplexers, as well as the output ports. *V_COMP_* is passed to the positive input of the comparator and *V_INTEG_* is passed to the negative input. The negative output of the comparator is sampled as *OUT*. The control word *m_y_* obtained in the tuning procedure could satisfy the following equation:

(17) $$C_{MIN}+m_{y}C_{UNIT}=\frac{I_{C}\times 0.5T}{V_{COMP}-V_{OFFSET}}$$

As discussed above, the target control word *m_TAGT_* satisfies Equation (14), which means the value of *m_TAGT_* is higher than *m_x_* and lower than *m_y_*. The mean value of *m_x_* and *m_y_* could be an approximation of *m_TAGT_*:

(18) $$m_{TAGT}\approx \frac{m_{x}+m_{y}}{2}$$

Note that if *V_OFFSET_* is extremely large and is comparable to *V_COMP_*, the accuracy of this strategy can be degraded, but the tuning accuracy is still improved by this strategy. In most cases, the value of *V_OFFSET_* is limited and is much lower than *V_COMP_*; therefore, Equation (18) would be a good approximation of *m_TAGT_*.

**Figure 4:**
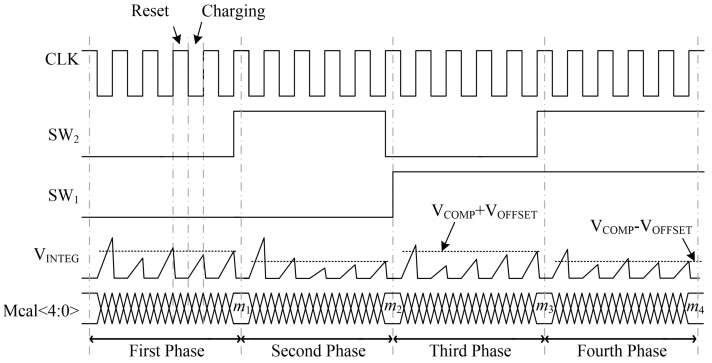
Transient waveforms of the proposed tuning circuit.

These two techniques are combined to improve the auto-tuning accuracy. As can be seen in Figure 4, the overall auto-tuning process includes four phases, and the time constant is calibrated once in each phase. There are four combinations for the values of *SW*_1_ and *SW*_2_, which are “0-0”, “0-1”, “1-0” and “1-1”. Each phase adopts one of these combinations. A successive approximation procedure is utilized to accelerate the tuning process. The digital block executes the successive approximation procedure to accelerate the tuning process. The process is described in the following four phases:

- For the first phase, *SW*_1_ and *SW*_2_ are both set to 0. The multiplexers pass *V_COMP_* to the negative input of the comparator and *V_INTEG_* to the positive input. The positive output of the comparator is sampled as *OUT*. The redundant *C*_BANK_ and transistor *MP*2 are cut off by *S*_1_ and *S*_2_. The control word is searched by means of the successive approximation procedure. The resultant value of the control word *Mcal*<4:0> is latched up as *m*_1_.
- For the second phase, *SW*_1_ stays 0 and *SW*_2_ switches to 1. The input ports of the comparator are reversed by the multiplexers, as well as the output ports. The same calibration procedure is repeated for a second time, and the resultant control word is latched up as *m*_2_.
- For the third phase, *SW*_1_ switches to 1 and *SW*_2_ switches to 0. In this phase, *MP*3 and the redundant *C_BANK_* take part in charging *V_INTEG_*. Two capacitor banks are charged by 2*I*_C_ at the same node. The connections of the multiplexers are the same as in phase 1. The control word *m*_3_ is obtained by a tuning procedure.
- For the fourth phase, *SW*_1_ and *SW*_2_ are both 1. *V_COMP_* is passed to the positive input of the comparator and *V_INTEG_* is passed to the negative input. The redundant part is involved as in phase 3. The calibration result is *m*_4_.

Once *m*_1_~*m*_4_ are obtained, a final control word can be inferred to avoid the influence of *C_P_* and *V_OFFSET_*. The final value of the control word *m_TAGT_* can be expressed as

(19) $$m_{TAGT}=\frac{\left(2m_{3}-m_{1}\right)+\left(2m_{4}-m_{2}\right)}{2}=m_{4}+m_{3}-\frac{m_{2}+m_{1}}{2}$$

The value of *m_TAGT_* is latched up and transferred to the capacitor bank in the slave circuit and the auto-tuning is finished. The master circuit is deactivated to reduce the power consumption and can be reactivated if needed.

**Figure 5:**
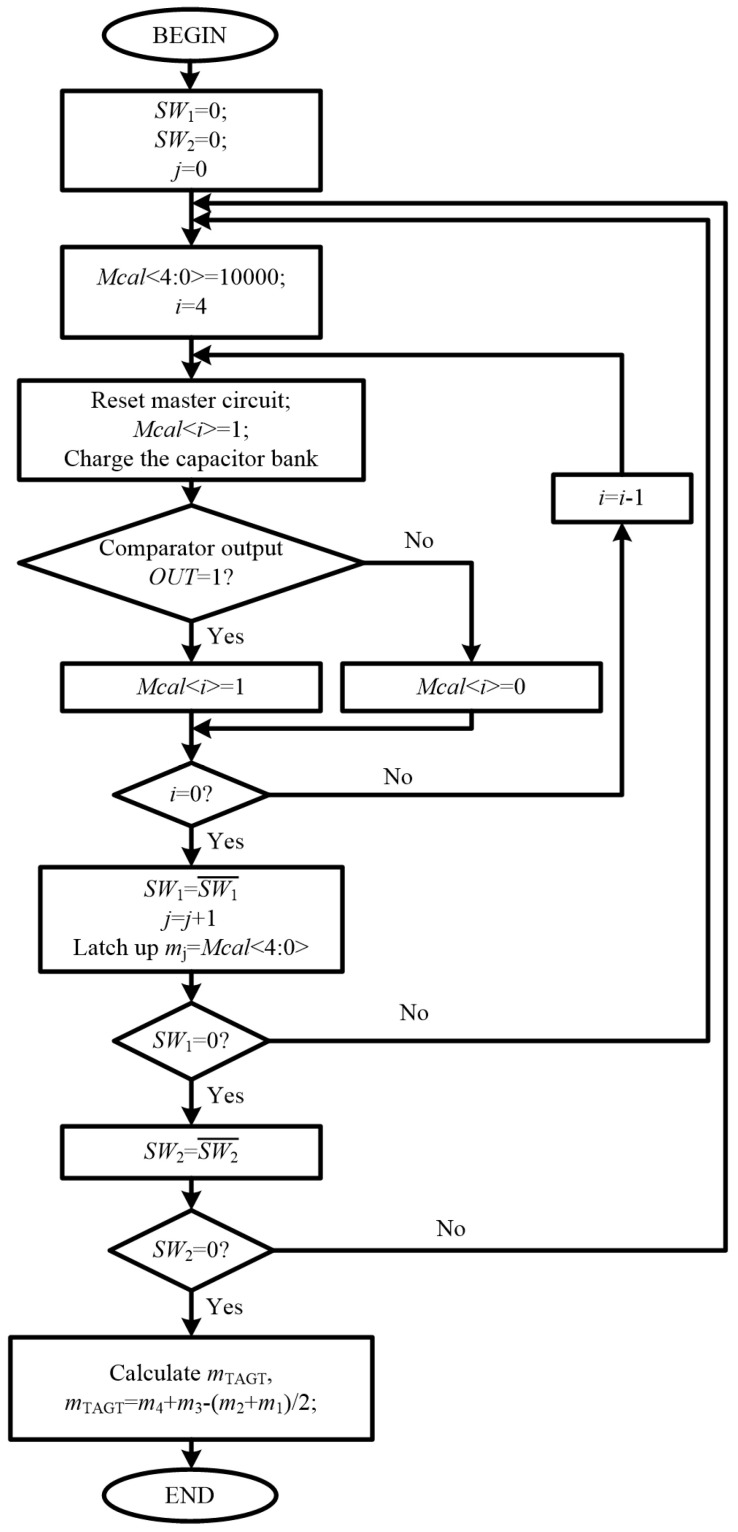
Flow chart of the four-phase tuning procedure.

Figure 5 illustrates the flow chart of the tuning procedure through successive approximation. *Mcal*<4:0> is set to “10000” in the beginning. *CLK* is 1 in the reset phase, the capacitor bank is discharged and *V_INTEG_* is set to be 0. The charging mode begins at the negative edge of *CLK*. *C_BANK_* is charged for a duration of *T*/2. Comparison is then performed between *V_INTEG_* and *V_COMP_*, and the comparison result is transferred to the digital block. If *V_INTEG_* is higher than *V_COMP_*, which means the present value of *C_BANK_* is lower than the nominal value, the output of comparator *OUT* is high; otherwise, *OUT* is low. The value of *Mcal*<4:0> is altered after each comparison. If *OUT* is high, set the most significant bit of *Mcal*<4:0> to be 1, otherwise set it to be 0. In both conditions, set the next bit to be 1 for the next reset-charge cycle. For example, if the output of the comparator in the first process is high, set *Mcal*<4:0> to be “11000” for the second reset-charge process, otherwise set it to be “01000”. Then repeat the reset-charge and comparison process. Move from the most significant bit to the least significant bit and apply the same operation on each bit. The value of *Mcal*<4:0> is registered after five reset-charge operations. This routine is performed four times under four different combinations of the values of *SW*_1_ and *SW*_2_. Four binary control words can be obtained, and the final control word is calculated by Equation (19) and transferred to the slave circuit.

The digital control word is sequentially increased or decreased by one least significant bit in the conventional tuning scheme; the longest tuning procedure may take 2*^n^*^−1^ cycles of the reset–charge operation. In this work, only *n* cycles are needed for the same tuning procedure due to successive approximation. Indeed, as a cost of diminishing nonidealities, both two-way comparing strategy and capacitor bank redundancy will double the tuning time. However, 4*n* cycles is still less than 2*^n^*^−1^ in applications where the control word is wider than five bits, which is usual when high tuning accuracy or a wide tuning range is demanded. Furthermore, the calibration process can be performed four times or only once according to the actual situation. In less critical applications, it is not necessary to excessively pursue high tuning accuracy; therefore, the calibration process can be performed only once after powering on, reducing the tuning time and the power consumption.

## 4. Circuit Design and Simulation Results

The proposed tuning circuit was designed under an SMIC 55 nm CMOS process, provided by Semiconductor Manufacturing International Corporation. In order to validate the auto-tuning circuit, a 4th-order low-pass active-RC filter was also designed as the circuit to be tuned.

### 4.1. Circuit Design

**Figure 6:**
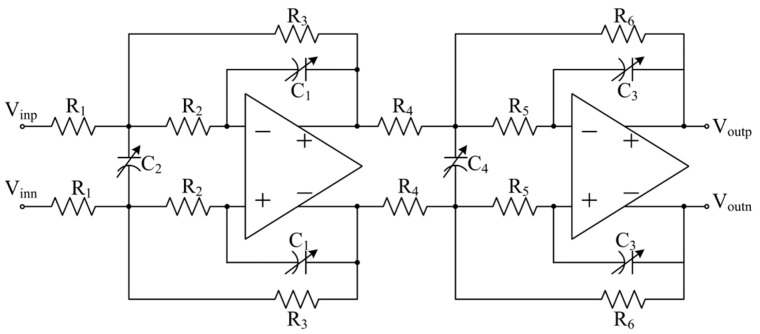
The 4th-order low-pass active-RC filter.

The digital block including SAR logic was realized using hardware description language. Two same capacitor banks with a 5-bit control word were applied in the circuit. The range of both capacitor banks were (50%, 150%) of the nominal capacitance. The comparator in the circuit is a conventional dynamic double-tail comparator, which shows high speed and power efficiency [32]. The 4th-order low-pass active-RC filter was cascaded by two MFB biquads, as shown in Figure 6. The cut-off frequency *f*_c_ for attenuation of 0.5 dB was designed to be 3 MHz, and the passband gain was 0 dB. The input IP3 was 17 dBm given a typical process corner, room temperature and nominal power supply. The capacitance values of the four different capacitors in the filter are designed as integer multiples of the capacitor bank in the calibration circuit; once the capacitor bank is tuned, the same capacitor banks can be used to form the capacitors in the filter.

Note that the designer should set the value of capacitor banks according to the power, area and tuning accuracy constraints, like in all the master–slave tuning techniques. A larger capacitance value of the capacitor bank will result in a higher tuning accuracy because it is closer to the real capacitance value required in the filter. However, the area and power consumption will be higher. A smaller capacitor bank will make the area and power consumption lower, but more capacitor banks would be used in the slave circuit; nonidealities such as coupling and parasitic capacitances will have a serious impact on the calibration accuracy.

**Figure 7:**
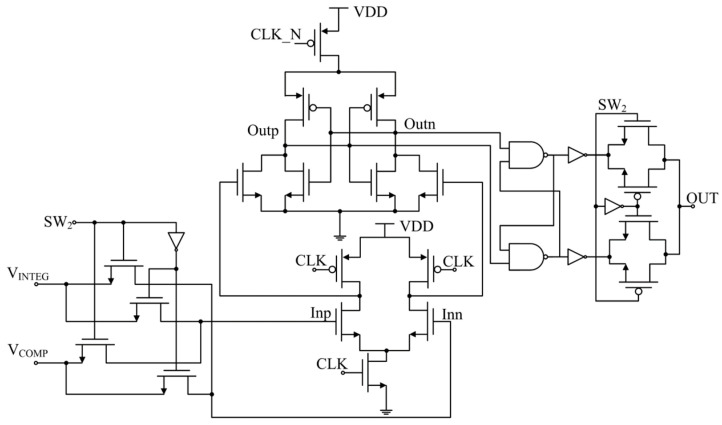
Comparator circuit.

The calibration accuracy could be affected if the output delay of the comparator is high; therefore, a dynamic comparator is adopted to achieve high speed and power efficiency. The comparator accompanied by the multiplexers is shown in Figure 7. This is a classic dynamic double-tail comparator. The output delay time of the dynamic comparator is much shorter than the clock period, leaving a large time margin for output sampling; therefore, the calibration accuracy is not affected. Under the control of a 1-bit signal *SW*_2_, the CMOS multiplexers could exchange the connection of two input nodes, helping to minimize the error induced by the DC offset as discussed earlier.

### 4.2. Simulation Results

Since the offset voltage is caused by random mismatches, the offset voltage of the dynamic comparator was investigated through Monte Carlo simulations. Process variation and mismatch were both considered in the simulations. As a result, input offset voltages up to 60 mV were observed. Therefore, an input offset voltage of 60 mV was applied to the comparator in simulations for illustration.

Post-simulations were carried out under PVT variations to validate the auto-tuning scheme. A voltage source of 60 mV were applied to model the effect of offset voltage. The nominal supply voltage VDD was 1.2 V. Eight corners were considered besides the typical corner. The temperature varied from −40 °C to 125 °C, and the supply voltage varied from 1.1 V to 1.3 V.

The simulated and calculated control words at one of the corners are listed in Table 1 as an example. The process corner was FF, supply voltage was 1.1 V and the temperature was 125 °C. Parasitic capacitance accounts for the difference between the control words obtained in phase 1 and phase 3. The difference between the control words obtained in phase 1 and phase 2 is induced by the offset voltage. As can be seen, the effects of these nonidealities have been minimized by the calculated control word.

**Figure 8:**
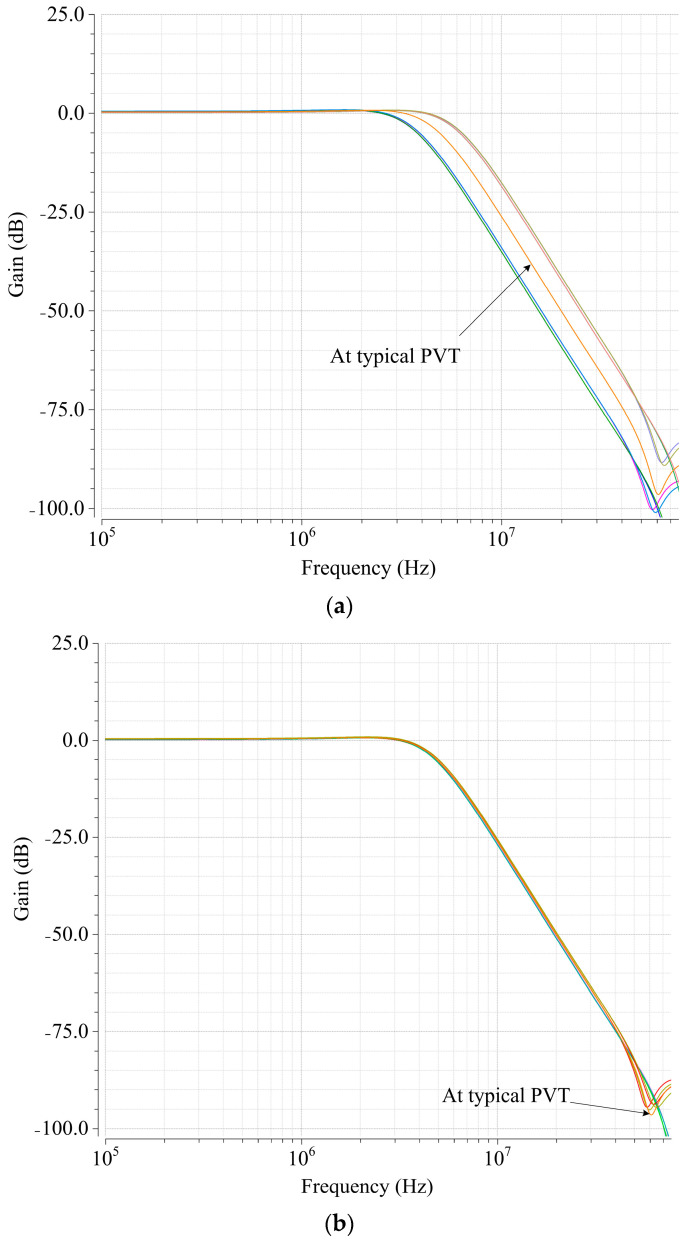
Simulated frequency response of the filter under PVT variations (**a**) before calibration and (**b**) after calibration.

Figure 8 shows the simulated frequency responses in all nine corners of the filter before and after RC time constant calibration. Obvious errors can be observed before the calibration. Table 2 shows the 0.5 dB cut-off frequency *f*_c_ at nine corners, whose first row indicates the result at a typical corner. The average tuning error was 2.21%, and the tuning error spans from 0.33% to 3.67% which includes the quantization error due to the discrete approach and other non-idealities. The whole tuning process took 3 µs, including sufficient time margin for digital block and general reset. The convergence time of the automatic calibration is determined by the number of bits of the binary control word. For certain bandwidth and resistance value, the capacitor bank can be binarily weighted by more bits or less bits. In other words, the designers need to trade off between convergence time and quantization error.

**Figure 9:**
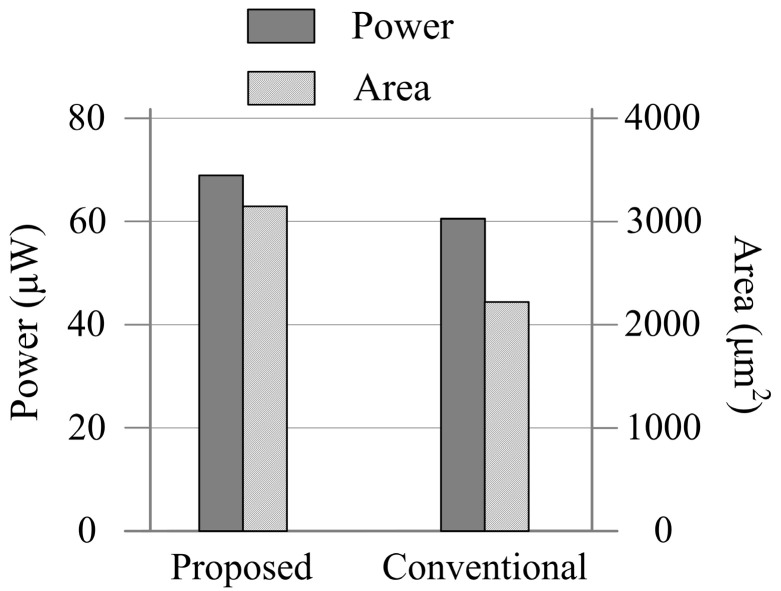
Circuit area and power consumption of the tuning circuits.

**Figure 10:**
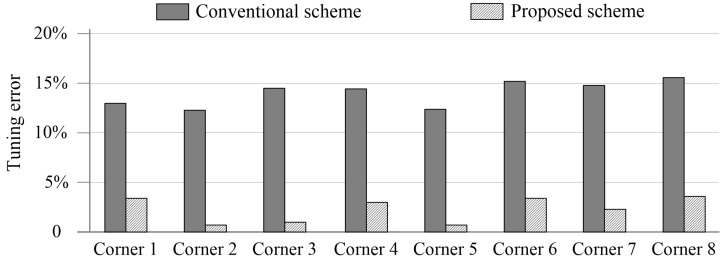
Tuning error at different corners.

The conventional scheme discussed in Section 2 was also designed for comparison. Figure 9 compares the total circuit area and average power consumption of the tuning schemes. Figure 10 shows the tuning error of the proposed scheme and conventional scheme at different corners. As can be seen, due to the parasitic capacitance and the offset voltage of the comparator, the conventional scheme shows a large tuning error which spans from 12.33% to 15.67%. However, the influences of these nonidealities have been minimized by the proposed scheme. The tradeoffs are that the proposed tuning scheme shows 13.7% higher power and 41% higher area consumption than the conventional scheme. As the tuning circuits are powered off after the tuning process, these tradeoffs are acceptable to applications that are not highly power-sensitive or area-sensitive. 

## 5. Conclusions

The accuracy of the RC time constant is critical for many continuous-time analog circuits. This paper reports a high-accuracy on-chip auto-tuning scheme to calibrate the RC time constant of integrated continuous-time filters. Two techniques are introduced to improve the tuning accuracy, namely the parasitic capacitance cancelation technique and the symmetric comparison technique. The tuning scheme is validated in 55 nm CMOS technology. Results show that the tuning error induced by parasitic capacitance and the DC offset can be reduced. The scheme is suitable for continuous-time systems where high tuning accuracy is demanded.

## Figures and Tables

**Table 1:** Digital control words in the tuning process under a process corner of FF, supply voltage of 1.1 V and temperature of 125 °C.

Digital Control Word	Value
*m*_1_ obtained in phase 1	10010
*m*_2_ obtained in phase 2	10011
*m*_3_ obtained in phase 3	10111
*m*_4_ obtained in phase 1	11000
Calculated *m_TAGT_*	11101

**Table 2:** The cut-off frequency under PVT variations.

Corners	Process Corner	Voltage (V)	Temperature (°C)	*f*_c_ before Calibration (MHz)	*f*_c_ after Calibration (MHz)	Tuning Error
Typical Corner	TT	1.2	27	3.00	-	-
Corner 1	FF	1.1	−40	4.00	3.10	3.33%
Corner 2	FF	1.1	125	3.98	3.02	0.67%
Corner 3	FF	1.3	−40	3.95	2.97	1.00%
Corner 4	FF	1.3	125	3.97	2.91	3.00%
Corner 5	SS	1.1	−40	2.27	2.99	0.33%
Corner 6	SS	1.1	125	2.27	2.90	3.33%
Corner 7	SS	1.3	−40	2.26	2.93	2.33%
Corner 8	SS	1.3	125	2.25	2.89	3.67%

## Data Availability

Data are contained within the article.
